# Nurturing *Global Health Action* through its first decade

**DOI:** 10.1080/16549716.2019.1569847

**Published:** 2019-02-07

**Authors:** Peter Byass, Nawi Ng, Stig Wall

**Affiliations:** Department of Epidemiology and Global Health, Umeå University, Umeå, Sweden

In 2008, the global publishing landscape for global health was markedly different from how it is now. The concept of open-access publishing across all disciplines was in its infancy, and many institutions and researchers were suspicious about paying publication fees, while nevertheless being content not to have to subscribe to journals. ‘Global Health’ as an academic discipline was a new evolution, emerging from the earlier concepts of ‘Tropical Medicine’ and ‘International Health’, in which a number of existing journals were still rooted. *Global Health Action* came tentatively into this space [1], and has tried to live up to Beaglehole and Bonita’s proposed definition of global health as ‘collaborative trans-national research and action for promoting health for all’ [2]. Unfortunately global health has not yet entirely succeeded in shedding the north–south axis underlying its antecedent paradigms [3]. Nevertheless, *Global Health Action* made encouraging progress in disseminating actionable science over its first five years [4].

Not surprisingly, other actors with complementary emphases have joined us in what has become a vibrant overall space of open-access global health publishing. For example, the *Journal of Global Health* launched in 2011 [5], *The Lancet Global Health* in 2013 [6] and *BMJ Global Health* in 2016 [7]. All of these, and other journals that overlap similar spaces, now provide a wide range of opportunities for publishing scientific advances in global health. Each journal has its own priorities and character: at *Global Health Action* we continue to emphasise science leading to implementation and action, and often comment editorially that manuscripts we receive lack the ‘action’ dimension. We continue to make distinctive contributions though three specific article types: PhD Reviews (in which recently defended PhD theses are distilled into review articles); Study Design articles (for describing the background and methods for major studies before they generate results); and Capacity Building articles (describing advances in global health research capacity).

**Figure 1.**
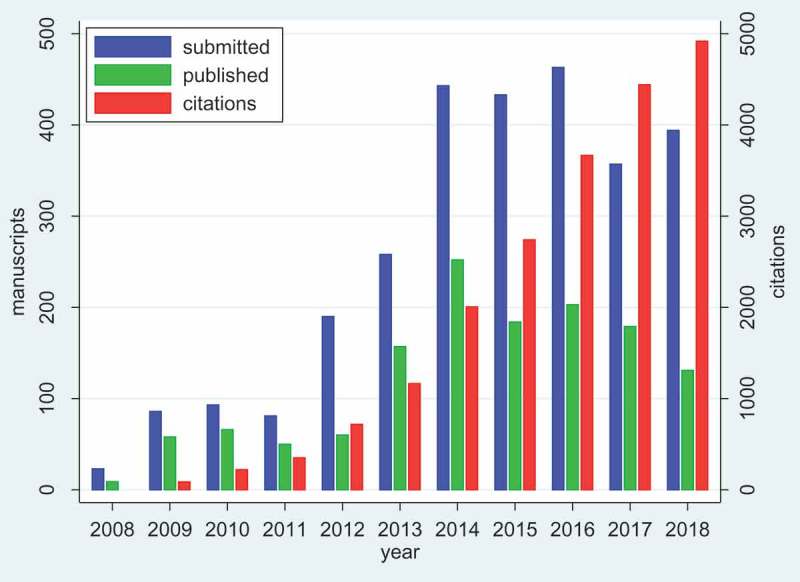
Numbers of manuscripts submitted and published by year in *Global Health Action*, with number of Google Scholar citations by year.

Tracking the progress and impact of a journal over time, despite the plethora of modern bibliometric tools, is not easy. Particularly for a journal like *Global Health Action*, where we hope to make an impression not only on the global scholarly community, but also on policy makers and implementers, we are interested in the use of our articles in the widest possible sense. We also recognise that global health is not a field in which articles are necessarily used and cited rapidly after publication. We find Google Scholar to be a useful tool, because it is not confined to the peer-reviewed literature. *Global Health Action* has its own Google Scholar public profile [8]. As of 31 December 2018, *Global Health Action* had accumulated 20,751 citations to its 1349 published papers (mean 15), with an h-index of 55 (meaning that 55 papers had 55 or more citations). Figure 1 shows the submissions, publications and Google Scholar citations by year since the journal began.

**Figure 2.**
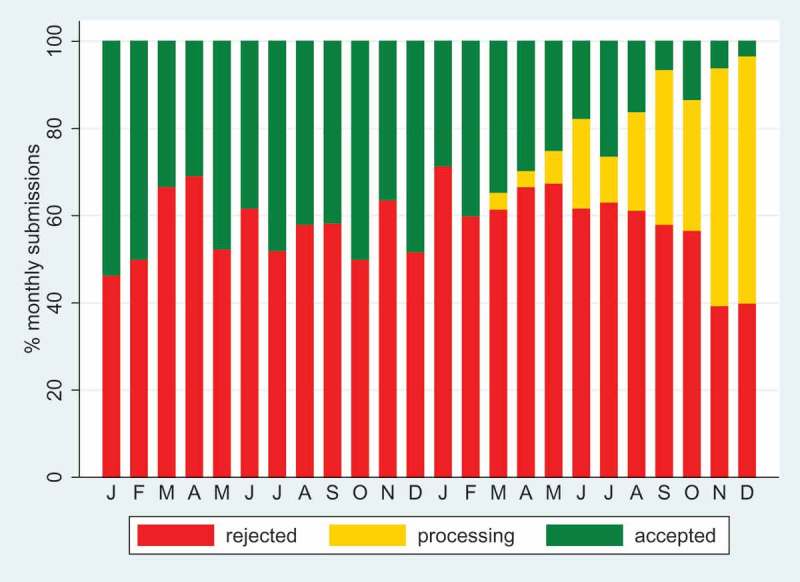
Editorial dynamics for *Global Health Action* as at 31 December 2018, covering 751 manuscripts submitted during 2017–18, by month.

As well as any journal’s long-term track record, recent performance is also important. At the end of 2016, our original publisher, Co-Action Publishing, was taken over by Taylor & Francis. On reflection, for the journal this was a similar process to a researcher replacing their computer: a mixture of short-term pain and longer-term gain. Having now completed two operational years with Taylor & Francis, the dynamics of our editorial performance are summarised in Figure 2, as of 31 December 2018. We received 357 submissions in 2017 and 394 in 2018; only 7% of these stayed with us for more than 6 months. Overall 435 manuscripts (57.9%) were rejected, 78 (10.4%) were still in progress at the end of 2018 and 238 (31.7%) were accepted. Mean time to rejection was 32 days, or to acceptance 118 days.

**Figure 3.**
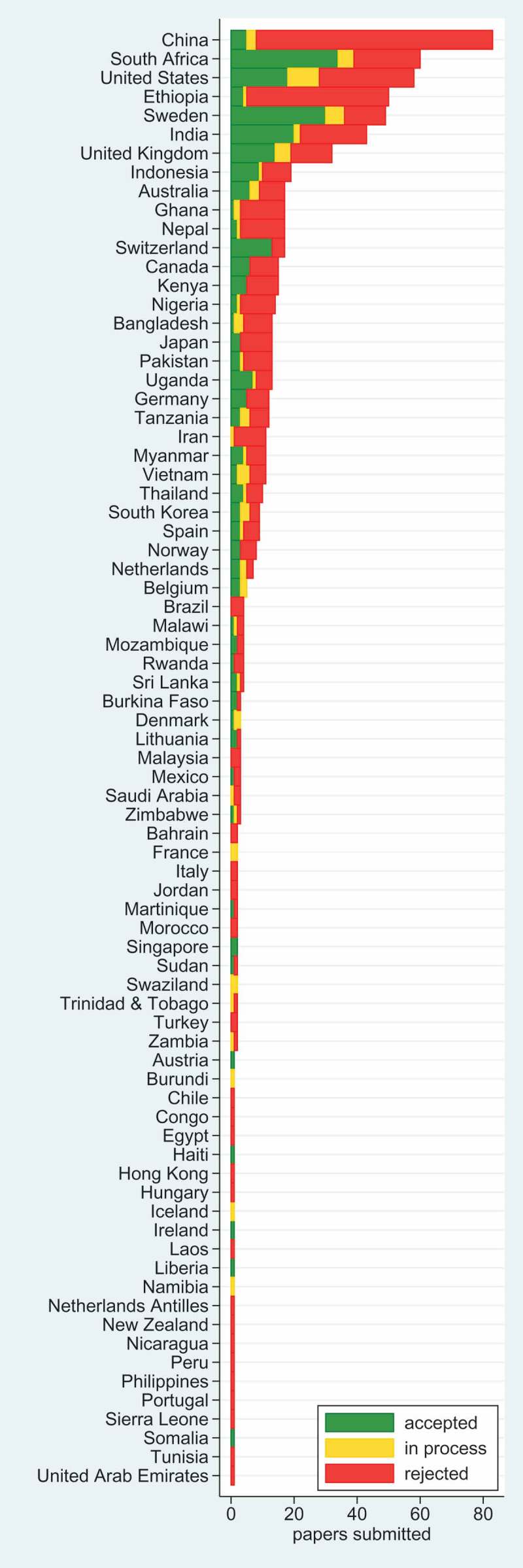
Country and publication status as at 31 December 2018 for 751 manuscripts submitted to *Global Health Action* during 2017–18.

Given the global nature of our subject matter, the geographical distribution of our material is a major interest. Figure 3 shows the same 751 manuscripts submitted during 2017–18 by country of origin (as chosen by authors at submission) and status at the end of 2018. Overall, 77 countries were represented. Unsurprisingly, China – with the world’s largest population and a well-established scientific infrastructure – was the largest contributor of manuscripts (83), although 90% of those were rejected. Manuscripts from Switzerland had the highest acceptance rate (76%), though that probably reflects the predominant international health organisations located there. Grouped by World Health Organization (WHO) region, the African region dominated our submissions (209 submissions, 64 accepted), followed by Europe (156 submissions, 79 accepted). South American countries were under-represented.

Our reflections on this decade of experience with *Global Health Action* (apart from the obvious fact that running a good international journal involves a lot of hard work) are that to a large extent we have succeeded in creating a valuable open-access space for global health science. We have established editorial practices that are both rigorous and reasonably fast, and many of our published articles have become highly cited. Above all, we are proud to receive many manuscripts from authors in low- and middle-income countries, a good proportion of which we are able to publish. This goes some way to pushing back the prevailing situation whereby the majority of published health research papers relate to population health in Europe and North America. In addition to individual papers, we have also collaborated with a range of international institutions and organisations in publishing special issues on important global health themes. As the global community works towards the United Nations’ Sustainable Development Goals and the World Health Organization renews global emphasis on ‘Health for All’, we trust that *Global Health Action* – through our readers, authors, reviewers, editors and publishers – will continue to play a significant role in disseminating the underlying global health science.
